# Case Report of Percutaneous Tract Seeding of Renal Pelvic Tumor: 8-Year Journey

**DOI:** 10.1089/cren.2016.0106

**Published:** 2016-11-01

**Authors:** Paulos Yohannes

**Affiliations:** Urology Health Center, Fremont, Nebraska.

**Keywords:** percutaneous tract seeding, renal TCC, percutaneous surgery

## Abstract

A 58-year-old female presented with renal colic and was found to have renal transitional cell carcinoma at the time of percutaneous surgery. She developed percutaneous tract seeding that clinically presented as subcutaneous skin nodules. After local treatment with surgical excision and radiation treatment, the patient developed retroperitoneal recurrence 5 years later. Percutaneous tract seeding is rare. There is no general consensus on prevention of tract seeding during percutaneous resection of renal urothelial tumors. Various recommendations from the literature are discussed.


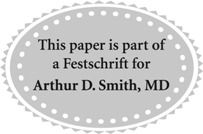


## Introduction

Tumor seeding along the percutaneous biopsy tract for solid renal tumors and during percutaneous endoscopic resection of transitional cell carcinoma (TCC) of the kidney has been reported. This article contains a rare case report of an 8-year long progression of high-grade TCC of the kidney incidentally found at the time of percutaneous renal stone surgery.

## Case Report

**FIG. 1.**
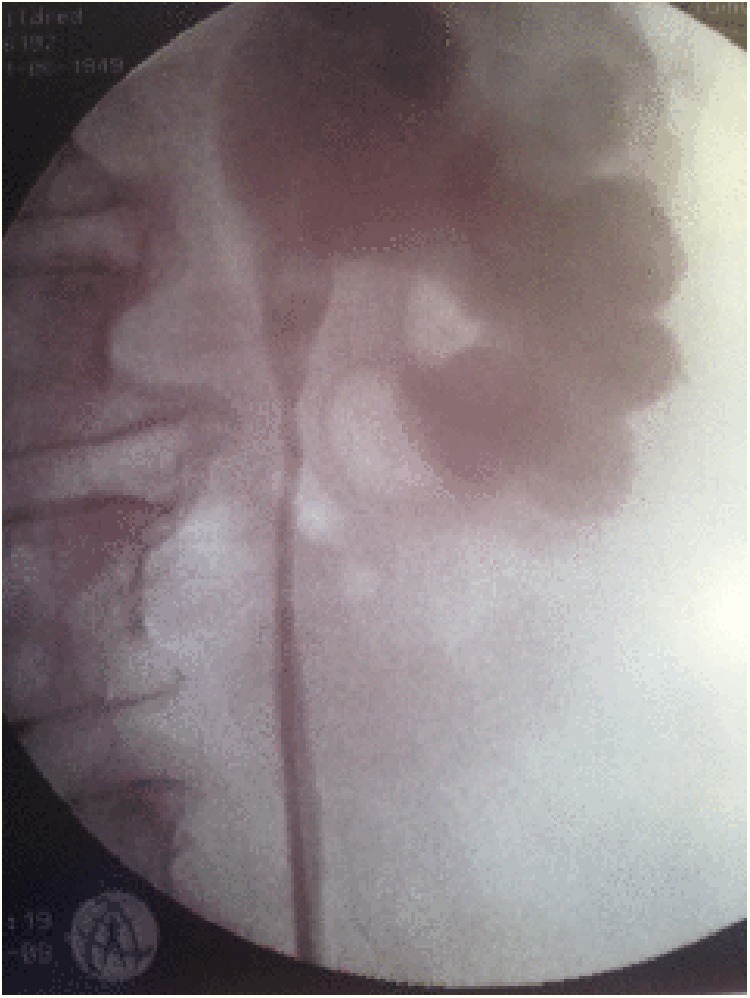
Left retrograde study showing megacalycosis.

**FIG. 2.**
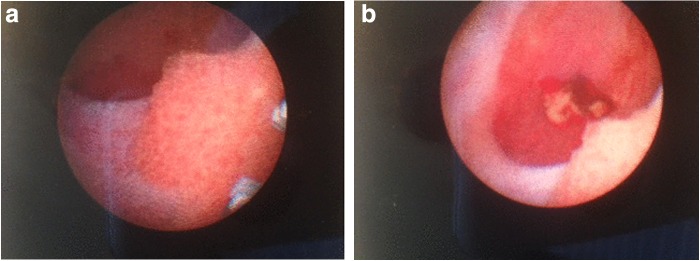
Percutaneous endoscopic view of renal TCC in mid pole calyx.

In June 2008, a 58-year-old female patient with history of breast cancer (s/p radical mastectomy and chemotherapy in 2000), kidney stones, and congenital megacalycosis of the left kidney developed renal colic secondary to large obstructing calculi, causing secondary ureteropelvic junction obstruction (Fig. 1). The patient underwent percutaneous stone extraction. During the procedure, a low-grade, papillary TCC of the lower pole calix was noted (Fig. 2a, b). The nephrostomy tube (NT) was left indwelling until the percutaneous tract matured and urine cleared, and 2 weeks later in July 2008, the patient was taken for repeat percutaneous endoscopy; there were no residual urothelial tumors noted in the kidney and the entire ureter, and the NT was removed. In October 2008, the patient developed gross hematuria and underwent resection of a bladder mass at the left ureteral orifice for high-grade TCC of the bladder with involvement of the lamina propria. Subsequently, left retrograde endoscopic evaluation of the kidney revealed a high-grade lesion within the middle calix.

In December 2008, the patient underwent laparoscopic radical nephroureterectomy with open excision of the left ureteral orifice. Surgical pathology analysis revealed high-grade, 2.6 cm TCC with microinvasion of the renal papilla (T3) and no invasion of perinephric fat. She received four cycles of carboplatinum and Gemza completed in April 2009. In July 2010, she presented with subcutaneous, palpable, painful nodules over the NT site and underwent wide skin excision; adjuvant 6000 cGy external beam radiation therapy was administered to the flank over a period of 8 weeks. In the following years, the patient developed numerous bladder tumor recurrences, which were managed with resection/fulgurations with maintenance intravesical chemotherapy.

**FIG. 3.**
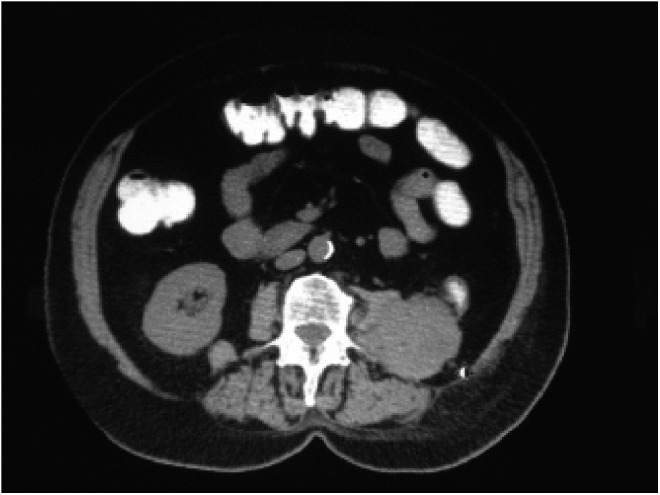
Left Retroperitoneal mass invading psoas muscle.

In 2015, the patient presented with severe back pain, malaise, fatigue, and upper gastrointestinal symptoms. Radiographic imaging studies showed 8.9 × 4.5 cm retroperitoneal mass involving the descending colon and quadratus lumborum (QL) near the region of the left renal fossa consistent with recurrent TCC (Fig. 3); there was no evidence of other systemic disease. The patient had developed congestive heart failure as a complication of chemotherapy in 2000 and was not a candidate for more systemic therapy. The patient underwent en bloc resection of mass, QL, and descending colon to relieve obstruction. Eleven months later, the patient developed a fatal stroke and died with widespread metastatic disease in July 2016.

## Discussion

Solitary metastatic nodule of the skin, as a natural progression of urologic cancer, is a rare clinical finding. Similarly, iatrogenic metastatic skin nodules in the management of urologic cancers are almost equally uncommon; these have been typically observed during percutaneous biopsy of papillary and clear cell renal carcinoma, upper tract urothelial carcinoma, and percutaneous resection of urothelial tumors of the kidney. Although image-guided percutaneous biopsy is a useful diagnostic clinical tool, it is associated with a small risk of tract seeding. Beyond diagnosis, percutaneous renal TCC resection as a treatment modality has become increasingly common, but even in experienced hands, it can result in percutaneous tract seeding. Urothelial tumor tract seeding is more common with high-grade lesions and has been reported at an incidence of 1.1% in high-volume academic centers.^1^

A recent review of the literature, including one by Huang et al. reviewing the safety and diagnostic accuracy of percutaneous biopsy in upper tract urothelial carcinoma, reveals only eight true cases of percutaneous tract recurrences at the access site after percutaneous resection since 1986.^2^ Furthermore, percutaneous NT insertion to relieve obstructive uropathy in patients with renal TCC who subsequently underwent nephroureterectomy led to recurrence within the NT tract in three patients. Finally, there have only been two reported cases of tract seeding after fine needle aspiration of renal TCC with 22- and 20-gauge needles.

The same authors report their experience of 26 procedures for 24 lesions and report no tract seeding.^1^ Similarly, Goel and coworkers report on 24 patients who underwent percutaneous resection for renal TCC. At a mean follow-up of 60 months, there were no cases of tract seeding. Although they routinely excise the percutaneous tract at the time of nephroureterectomy, all excised tracts were free of tumor in their series.^3^ Patel et al. have also reported on their experience in long-term outcome after percutaneous treatment in 26 patients, no percutaneous tract seeding was noted after irradiating the percutaneous tract with iridium in 12 patients and high-dose external radiation in 12.^4^

Finally, Rastinehad and collegues in their largest series to date report on their experience with the percutaneous approach in 89 patients (133 renal units) between 1985 and 2005. At a mean follow-up of 61 months, there was only one case of percutaneous tract seeding. The incidence of local recurrence was 33%, with an overall survival of 68%. They assert that there is no overall benefit for adjuvant BCG treatment with regard to disease recurrence, interval to recurrence, and progression of disease.^1^

Although several recommendations have been made to minimize risk of tumor seeding during percutaneous needle biopsy of solid organ tumors, such as the use of coaxial biopsy sheath and minimizing the number of needle passes, there is no common consensus on minimizing or preventing tract seeding at the time of percutaneous surgery.^1^ Some of the recommendations include using a large Amplatz sheath to decrease intrarenal pressure and irrigation of percutaneous tract with chemotherapy solutions.

In the absence of systemic disease progression beyond the skin nodules, the role percutaneous tract seeding plays, after local treatment, as a nidus for future systemic progression of disease remains unclear. Regardless, patients should be made aware of the potential risk, and the potential role for chemotherapy in this instance should be revisited.

Although tumor seeding during planned percutaneous urothelial lesion resection is rare, incidental finding of TCC during stone surgery creates a greater dilemma. The radiographic imaging for stone disease precludes the use of contrast, enabling TCC to go unnoticed, and percutaneous access to the calix of interest will be dictated by the location of the stone rather than the location of the tumor. What then is the common factor among those cases in whom percutaneous tract seeding has been diagnosed? Several questions remain unanswered on how to minimize this risk, especially in cases in whom TCC is incidentally discovered at the time of percutaneous renal stone surgery, a typical scenario in which patient selection on the basis of tumor grade is not possible. In the latter case, should complete resection be undertaken at the time of lithotripsy? Should the NT be left in longer? What is the role of the immediate use of hemostatic agents in lieu of NT to seal the renal parenchyma? Would immediate ureteral stenting help in promoting urinary drainage? As urothelial tumors are radiosensitive, is there a role for adjuvant radiation treatment to the kidney and/or percutaneous tract? Which is the ideal chemotherapy agent to be used for percutaneous irrigation, and for how long?

## Conclusion

Iatrogenic percutaneous tract seeding of renal TCC is a rare event in the literature and may be under-reported. Surgeons should be more vigilant and take aggressive precautions to minimize morbidity and, if possible, prevent disease progression. Unfortunately, because of its rarity, standardized protocols and recommendations on preventing tract recurrence are not available.

## Abbreviations Used

TCC: transitional cell carcinoma
NT: nephrostomy tube
